# Association Between Physical Activity Intensity and the Risk for Depression Among Adults From the National Health and Nutrition Examination Survey 2007–2018

**DOI:** 10.3389/fnagi.2022.844414

**Published:** 2022-05-27

**Authors:** Donghui Yang, Ming Yang, Jianjun Bai, Yudiyang Ma, Chuanhua Yu

**Affiliations:** ^1^Department of Epidemiology and Biostatistics, School of Public Health, Wuhan University, Wuhan, China; ^2^Department of Medical Affairs, The First Affiliated Hospital of Xiamen University, School of Medicine, Xiamen University, Xiamen, China

**Keywords:** depression, vigorous physical activity (VPA), moderate-to-vigorous physical activity (MVPA), intensity, National Health and Nutrition Examination Survey (NHANES)

## Abstract

**Objective:**

Whether vigorous physical activities (VPA) bring additional benefits to depression prevention in comparison with moderate physical activity (MPA) remains unclear. The aim of this study was to find the correlation between the proportion of VPA to moderate-to-VPA (MVPA) (a combination of VPA and MPA) and the risk for depression, as well as to explore whether correlations differ among subgroups separated by age and sex.

**Methods:**

The data originating from the National Health and Nutrition Examination Survey (NHANES) 2007–2018 were applied. The total amount of PA per week was obtained by multiplying frequency and duration. The proportion of VPA to MVPA was obtained among the participants who performed any MVPA. Depression was set for those who scored 10 and above in the Patient Health Questionnaire-9 (PHQ-9). The odds ratios (ORs) and 95% confidence intervals (95% CIs) for depression were evaluated using logistic regression.

**Results:**

Among 26,849 participants of this study, only 12,939 adults were found with any MVPA, in which 748 participants with depression were detected. Logistic regression was conducted among 12,939 participants. The participants with higher than 66.7–100% of MVPA as VPA were inversely correlated with a 30% (OR = 0.70, 95% CI = 0.50, 0.99) lower risk for depression. The subgroup analyses revealed that significant correlations were only found in men and those aged 45 years and above.

**Conclusion:**

This study suggested that a higher proportion of VPA to MVPA might be correlated with a lower risk for depression in men and those aged 45 years and above. Besides the recommendation, adults should perform 150 min MVPA per week, more time should be spent in performing VPA in MVPA among men and older adults.

## Introduction

Depression refers to a common and growing global mental health issue (Park and Zarate, 2019). The World Health Organization (WHO) has suggested that people with depression reach over 300 million globally, which accounts for 4.4% of the world's population (Estimates, 2017). The estimated lifetime prevalence of depression is 21% for women and 11–13% for men (Kessler et al., 2003; Belmaker and Agam, 2008). Depression generated heavy health burden studies, ranking the largest contributor to disability worldwide (Estimates, 2017). It could cause huge loss of health correlated with cardiovascular disease (Elderon and Whooley, 2013), diabetes (Roy and Lloyd, 2012), and cancer (Bortolato et al., 2017). Furthermore, depression has been reported as the major cause of suicide, and it is currently one of the top 10 causes of death in the United States (National Center for Health Statistics, United States, 2017). Thus, the onset of depression should be reduced.

Numerous studies reported risk factors for the development and progression of depression (e.g., lifestyle factors and psychophysiological and psychosocial determinants; Munoz et al., 2010; Lopresti et al., 2013; Tuithof et al., 2018). Physical activity (PA) has been reported as a vital modifiable factor for depression prevention. Several previous studies have demonstrated that PA was correlated with a lower risk for depression (Schuch et al., 2018; Dishman et al., 2021). However, benefits of depression prevention may vary with the intensity of PA. WHO recommends that adults should perform 150–300 min moderate PA (MPA) per week, 75–150 min vigorous PA (VPA) per week, or 150–300 min moderate-to-VPA (MVPA) per week, i.e., an equivalent combination of VPA and MPA (Bull et al., 2020). The assumption relating to the PA guidelines is that VPA may be associated with higher health benefits than MPA. A previous study has explored the correlation between physical intensity and depression, and it was reported that depressive symptoms increased with the intensity of PA decreasing (Lampinen et al., 2000). In addition, another study also demonstrated that VPA had the lower odds ratio (OR) for more severe depression compared with MPA (Mumba et al., 2021). Currier et al. supported the above findings and suggested that a lower risk for depression was also observed when MPA was substituted with VPA at any level (Currier et al., 2020). However, as demonstrated by a study based on the Australian Longitudinal Study on Women's Health, performing VPA did not bring a significant additional benefit to depression, except at a very high level of PA (Pavey et al., 2013). Although a considerable number of studies have investigated the association between PA and the risk for depression, it remained unclear whether VPA offered additional benefits than MPA.

Accordingly, to verify whether VPA provides additional benefits, the proportion of VPA to MVPA was calculated, and the correlation between the proportion and the risk for depression was investigated based on data from the National Health and Nutrition Examination Survey (NHANES) 2007–2018. Furthermore, it was explored whether correlations differ between age and sex in this study.

## Materials and Methods

### Study Population

Data of this study originated from NHANES conducted by the National Center for Health Statistics (NCHS) of the CDC. In brief, NHANES was a nationally representative cross-sectional survey and used a multistage probability sampling design to collect information regarding health and nutritional status in the US. More detailed contents of NHANES have been previously published elsewhere (CDC, 2017). NHANES was approved by the NCHS Research Ethics Review Board. The respective survey participant provided informed consent. Information in this study was obtained from this publicly available and deidentified NHANES database so that this study was exempt from the Institutional Review Board review.

A total of 34,525 participants had complete data regarding VPA and MPA in six cycles from 2007 to 2018 in NHANES. Among 34,525 participants, we excluded 3,510 participants without a complete estimation of depression status. Furthermore, we excluded 4,166 participants with missing data regarding marital status, educational level, family income, body mass index (BMI), smoking status, and drinking status. Finally, this study included 26,849 participants, consisting of 13,910 participants not taking MVPA and 12,939 participants taking MVPA.

### Ascertainment of Depression Status

Depressive symptoms in the past 2 weeks were measured using the Patient Health Questionnaire-9 (PHQ-9), a well-validated instrument to evaluate the depression status (sensitivity: 88%; specificity: 88%; Kroenke et al., 2001). The PHQ-9 questionnaire contained nine items (i.e., anhedonia, depressed mood, sleep disturbance, fatigue, appetite changes, low self-esteem, concentration problems, psychomotor disturbances, and suicidal ideation), with the respective item scoring 0 (“not at all”), 1 (“several days”), 2 (“more than half the Days”), and 3 (“nearly every day”). Participants scoring 10 and above were considered to suffer from depression (Kroenke et al., 2001).

### Evaluation of Physical Activity

Information regarding PA from 2007 to 2018 was acquired in accordance with the World Health Organization Global Physical Activity Questionnaire (Armstrong and Bull, 2006). Participants were asked the following questions: “Do you do any vigorous-intensity sports, fitness, or recreational activities that cause large increases in breathing or heart rate like running or basketball for at least 10 min continuously?” and “Do you do any moderate-intensity sports, fitness, or recreational activities that cause a small increase in breathing or heart rate such as brisk walking, bicycling, swimming, or golf for at least 10 min continuously?” When they answered yes to the respective question, further questions about the frequency and duration of PA were inquired. The frequency of MPA/VPA was measured in accordance with the question “In a typical week, on how many days do you do moderate-intensity/vigorous-intensity sports, fitness or recreational activities?” Duration of MPA/VPA was measured based on the question “How much time do you spend doing moderate-intensity/vigorous-intensity sports, fitness or recreational activities on a typical day?”

The total amount of MPA and VPA was obtained by multiplying frequency and duration, accounting for intensity, MVPA (min/week) = MPA (min/week) + [2 × VPA (min/week)]. Among the participants performing any MVPA, we obtained the proportion of VPA to MVPA as follows: VPA × 2/MVPA × 100%. The proportion of VPA to MVPA was categorized as 0–33.3%, >33.3–66.7%, and >66.7–100.0%.

### Covariates

The information regarding covariates was collected through examination and questionnaire review. The covariates consisted of demographical factors (e.g., age, sex; race/ethnicity, marital status, education level, and family income) and lifestyle factors (e.g., BMI, drinking status, and smoking status). Race/ethnicity was categorized as four groups, including Hispanic, non-Hispanic white, non-Hispanic black, and other non-Hispanic. Marital status was categorized as married or living with partner; widowed, divorced, or separated; and never married. Educational level was divided into three levels, including < high school, high school, and >high school. Family poverty to income (PIR) threshold fell into three levels, including 0.0–1.0, 1.1–3.0, and >3.0. BMI, calculated as weight (kg) divided by height (m) squared, threshold fell into three levels, including <25, 25.0–29.9, and ≥30 kg/m^2^. Smoking status was divided into three groups, including never smoker, former smoker, and current smoker. Drinking status was categorized into three groups, including never drinker, former drinker, and current drinker. According to the WHO guidelines, participants were categorized into two groups whether meeting the WHO guidelines (≥150 min MVPA/week) or not.

### Statistical Analysis

Impacted by the complex design in NHANES, all analyses in this study considered sample weights, clustering, and stratification. Categorized variables were expressed as frequency with weighted percentage. Logistic regression was built to evaluate the ORs and 95% confidence intervals (CIs) for the correlation between the proportion of VPA to MVPA and depression. In this study, correlations between the proportion of VPA to MVPA and the risk for depression were examined in the model adjusted for multivariate, consisting of age (<45 years and ≥45 years), sex (men and women), race/ethnicity (Hispanic, non-Hispanic white, non-Hispanic black, and other non-Hispanic), marital status (married or living with partner; widowed, divorced, or separated; and never married), educational level (< high school, high school, and >high school), PIR (0.0–1.0, 1.1–3.0, and >3.0), BMI (<25.0, 25.0–29.9, and ≥30.0 kg/m^2^), smoking status (never smoker, former smoker, and current smoker), drinking status (never drinker, former drinker, and current drinker), and meeting PA guideline (yes or no). Subgroups analyses were conducted to evaluate the correlation between proportion and depression among groups separated by age (<45 years and ≥45 years) and sex (men and women).

In addition, the robustness of the results of this study was evaluated through sensitivity analyses. First, the E-value was adopted to evaluate the strength of the correlations, on the risk ratio scale, of an unmeasured confounder with both exposure and outcome needed to explain away the observed correlations (VanderWeele and Ding, 2017). Second, among the 31,015 participants (14,847 with MVPA and 16,168 without MVPA) with complete information regarding PA and depression, there were missing values for drinking status (*n* = 70; 0.2%), smoking status (*n* = 303; 1.0%), BMI (*n* = 326; 1.1%), marital status (*n* = 1,110; 3.6%), educational level (*n* = 1,116; 3.6%), and PIR (*n* = 2,840; 9.2%). Analysis was based on multiple imputed data regarding missing values.

Data analyses were conducted using Stata version 15. A 2-sided *P* < 0.05 was considered statistically significant.

## Results

A total of 26,849 adults were included in this study (13,213 men and 13,636 women), including 2,501 participants with depression and 24,348 participants without depression. Table 1 lists the basic characteristic of participants in this study. The estimation result showed that 13,910 participants did not perform MVPA in a week, accounting for 45.47% of total participants. Among the 12,939 adults reported with any MVPA, the proportion of VPA to MVPA showed the following distributions: over half of the participants (53.26%) were found with 0 to 33.3% of VPA, 15.11% were reported to be higher than 33.3–66.7% of VPA, and 31.63% were found to be more than 66.7% of VPA, respectively. Participants who were younger, men, non-Hispanic white, and married, with a higher educational level, with a high-income level, with a normal BMI (<25.0 kg/m^2^), with current alcohol assumption, and with no smoking history were found to be more likely to have a higher proportion of MVPA as VPA.

**Table 1:** Basic characteristics of 26,849 participants in this study from NHANES 2007–2018.

**Variables**	**No MVPA (%)**	**Proportion of VPA to MVPA**		
		**≥0.0 and ≤33.3 (%)**	**>33.3 and ≤66.7 (%)**	**>66.7 and ≤100.0 (%)**
Total	13,910 (45.47)	7,181 (53.26)	1,846 (15.11)	2,912 (31.63)
**Age, years**				
<45	4,774 (38.12)	2,626 (38.65)	1,156 (61.28)	2,670 (68.10)
≥45	9,136 (61.88)	4,555 (61.35)	690 (38.72)	1,242 (31.90)
**Sex**				
Men	6,492 (46.11)	3,299 (44.76)	1,051 (57.75)	2,371 (57.95)
Women	7,418 (53.89)	3,882 (55.24)	795 (42.25)	1,541 (42.05)
**Race/ethnicity**				
Hispanic	3,791 (15.92)	1,470 (10.01)	418 (13.39)	859 (12.94)
Non-Hispanic white	5,741 (64.80)	3,503 (74.44)	793 (69.08)	1,547 (67.14)
Non-Hispanic black	3,059 (12.16)	1,331 (8.40)	363 (9.34)	892 (11.27)
Other Non-Hispanic	1,319 (7.12)	877 (7.15)	272 (8.19)	614 (8.65)
**Marital status**				
Married or living with partner	8,235 (62.85)	4,447 (66.57)	1,102 (64.99)	2,170 (58.84)
Widowed, divorced, or separated	3,584 (21.88)	1,647 (19.15)	263 (11.07)	501 (11.41)
Never married	2,091 (15.27)	1,087 (14.27)	481 (23.95)	1,241 (29.74)
**Educational level**				
< high school	4,371 (22.54)	1,190 (10.05)	183 (5.77)	399 (6.16)
High school	3,599 (27.90)	1,615 (22.48)	303 (15.21)	656 (14.57)
>high school	5,940 (49.56)	4,376 (67.47)	1,360 (79.02)	2,857 (79.26)
**Family income-poverty ratio**				
0.0–1.0	3,630 (18.77)	1,235 (10.92)	287 (9.92)	630 (11.19)
1.1–3.0	6,436 (41.55)	2,908 (33.53)	611 (27.07)	1,369 (28.74)
>3.0	3,844 (39.68)	3,038 (55.56)	948 (63.02)	1,913 (60.07)
**BMI, kg/m** ^**2**^				
<25.0	3,435 (24.08)	2,024 (28.25)	640 (35.34)	1,497 (40.41)
25.0–29.9	4,390 (30.85)	2,375 (33.31)	632 (36.35)	1,370 (35.09)
≥30.0	6,085 (45.07)	2,782 (38.44)	574 (28.31)	1,045 (24.50)
**Drinking status**				
Never drinker	2,163 (12.18)	939 (10.11)	147 (6.77)	366 (6.75)
Ever drinker	3,137 (19.77)	1,208 (13.86)	198 (8.80)	403 (8.61)
Current drinker	8,610 (68.06)	5,034 (76.03)	1,501 (84.42)	3,143 (84.63)
**Smoking status**				
Never smoker	7,048 (50.01)	3,926 (54.33)	1,188 (64.24)	2,594 (66.84)
Ever smoker	3,482 (25.06)	1,967 (28.33)	363 (21.74)	760 (20.85)
Current smoker	3,380 (24.93)	1,288 (17.33)	295 (14.02)	558 (12.31)
**Meeting physical activity guideline**				
No	13,910 (100.00)	3,493 (49.69)	98 (5.14)	382 (8.72)
Yes	0 (0.00)	3,688 (50.31)	1,748 (94.86)	3,530 (91.28)
**Depression**				
No	12,157 (88.15)	6,690 (93.62)	1,762 (96.26)	3,739 (96.43)
Yes	1,753 (11.85)	491 (6.38)	84 (3.74)	173 (3.57)

*NHANES, National Health and Nutrition Examination Survey; VPA, vigorous physical activity; MVPA, moderate-to-vigorous physical activity; BMI, body mass index*.

*The NHANES used complex design. Weight was taken into consideration. Categorized variable was described as frequency and weighted percentage*.

After the multivariate adjustment for sociodemographic factors and lifestyle factors in logistic regression, a higher proportion of VPA to MVPA was found to be correlated with a lower risk for depression. Table 2 lists ORs with 95%CIs of the respective covariates. Demographic risk factors for depression consisted of women, lower educational level, and income, while marriage was indicated as a protective factor. Participants reporting over 66.7–100% of MVPA as VPA were found to be inversely correlated with the risk for depression (OR = 0.70, 95% CI = 0.50, 0.99). Furthermore, the magnitude of unmeasured confounding required to clarify this inverse correlation was 2.21 between proportions with higher than 66.7–100% of MVPA as VPA and depression (Supplementary Figure 1).

**Table 2:** ORs and 95% CIs for depression of the proportion of VPA to MVPA and other covariates.

**Variables**	**PHQ-9 ≥10**		**ORs (95 CIs)**
	**Yes**	**No**	
**Age, years**			
<45	400	6,052	1.00 (reference)
≥45	348	6,139	0.87 (0.68, 1.13)
**Sex**			
Men	301	6,420	1.00 (reference)
Women	447	5,771	1.54 (1.25, 1.89)
**Race/ethnicity**			
Hispanic	192	2,555	1.00 (reference)
Non-Hispanic white	312	5,531	0.94 (0.71, 1.26)
Non-Hispanic black	163	2,423	0.85 (0.65, 1.11)
Other Non-Hispanic	81	1,682	1.06 (0.75, 1.50)
**Marital status**			
Married or living with partner	323	7,396	1.00 (reference)
Widowed, divorced, or separated	224	2,187	1.82 (1.40, 2.37)
Never married	201	2,608	1.89 (1.44, 2.47)
**Educational level**			
< high school	194	1,578	1.00 (reference)
High school	164	2,410	0.73 (0.54, 1.00)
>high school	390	8,203	0.67 (0.48, 0.94)
**Family income-poverty ratio**			
0.0–1.0	251	1,901	1.00 (reference)
1.1–3.0	319	4,569	0.61 (0.49, 0.77)
>3.0	178	5,721	0.40 (0.31, 0.53)
**BMI, kg/m** ^**2**^			
<25.0	215	3,946	1.00 (reference)
25.0–29.9	195	4,182	0.87 (0.65, 1.18)
≥30.0	338	4,063	1.56 (1.18, 2.05)
**Drinking status**			
Never drinker	83	1,369	1.00 (reference)
Ever drinker	135	1,674	1.45 (0.96, 2.18)
Current drinker	530	9,148	1.10 (0.77, 1.57)
**Smoking status**			
Never smoker	334	7,374	1.00 (reference)
Ever smoker	181	2,909	1.36 (1.02, 1.82)
Current smoker	233	1,908	2.08 (1.58, 2.74)
**Meet physical activity guideline**		
No	300	3,673	1.00 (reference)
Yes	448	8,518	0.78 (0.59, 1.03)
**Proportion of VPA to MVPA**			
≥0.0 and ≤ 33.3 (%)	491	6,690	1.00 (reference)
>33.3 and ≤ 66.7 (%)	84	1,762	0.76 (0.55, 1.05)
>66.7 and ≤ 100.0 (%)	173	3,739	0.70 (0.50, 0.99)

*PHQ-9, Patient Health Questionnaire-9; ORs, odds ratios; CIs, confidence internals; BMI, body mass index; VPA, vigorous physical activity; MVPA, moderate-to-vigorous physical activity*.

**Figure 1:**
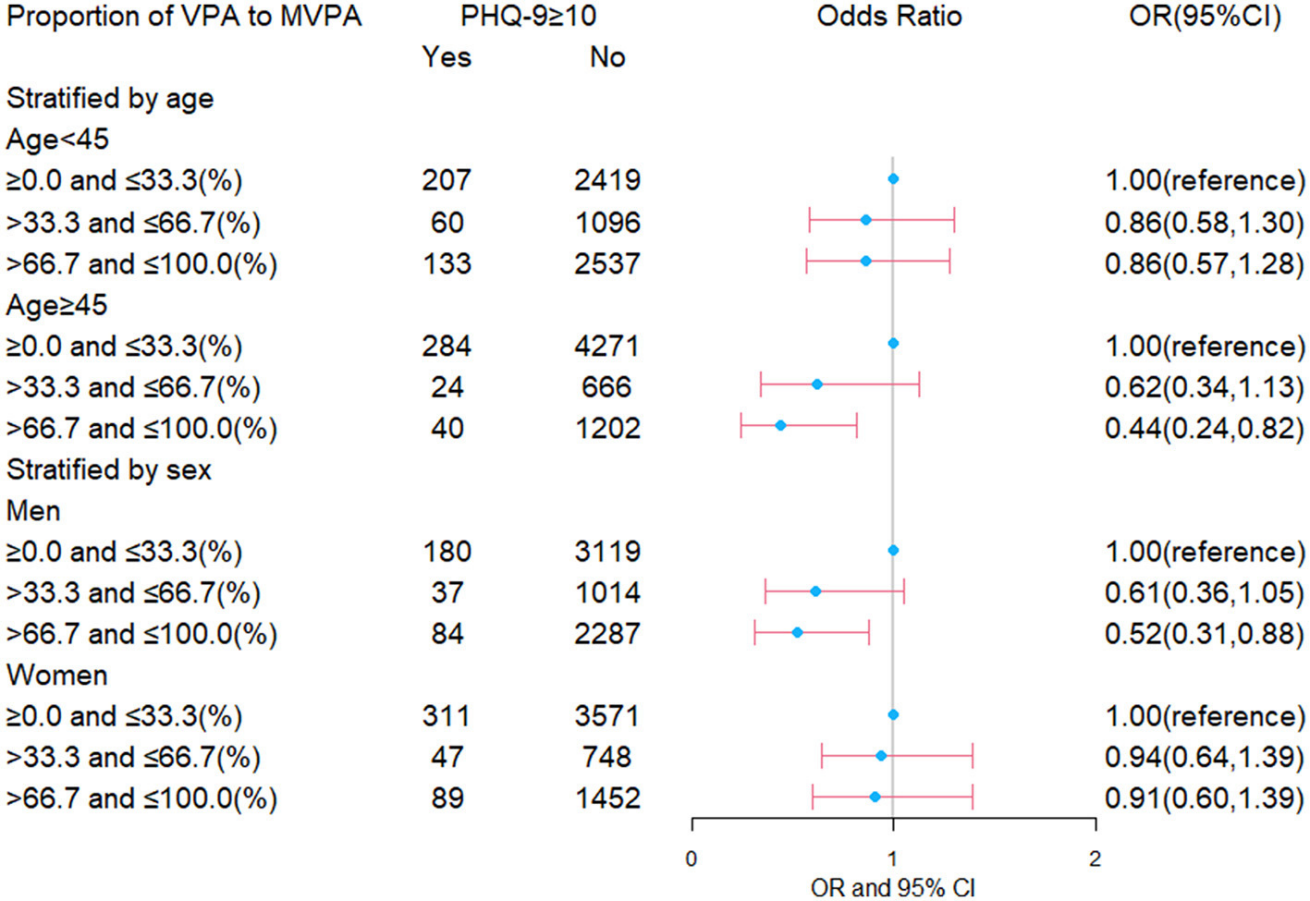
Correlation between the proportion of VPA to MVPA and the risk for depression among groups separated by age and sex. VPA, vigorous physical activity; MVPA, moderate-to-vigorous physical activity; PHQ-9, Patient Health Questionnaire-9; OR, odds ratio; CI, confidence internal.

Furthermore, Figure 1 presents the results in subgroups analysis stratified by age and sex. Correlations differed between age and sex in this study. When the analysis was conducted among groups separated by age, no significant correlation was found between proportion and depression among those aged less than 45 years. In contrast, among those aged 45 and above, participants performing over 66.7–100% of MVPA as VPA were found to be correlated with a 56% (OR = 0.44, 95% CI = 0.24, 0.82) lower risk for depression. For analysis among groups separated by sex, men with higher than 66.7% of MVPA as VPA were found to have a significantly lower risk for depression, whereas no significant correlation was observed in women. OR for depression was 0.52 (95% CI = 0.31, 0.88) for men with more than 66.7–100% of MVPA as VPA.

Analysis based on multiple imputed data found a similar correlation with analysis on complete data (Supplementary Figure 2). Furthermore, there was statistical significance in all proportion groups.

## Discussion

As revealed by the analyses conducted among US adults from 2007 to 2018 by NHANES, the participants with a higher proportion of VPA to MVPA might be correlated with a lower risk for depression. The analysis stratified by age and sex suggested that a lower risk for depression was primarily found among men and those aged 45 years and above. No significant correlations were found between proportion and depression in women and those aged <45 years.

About 9.3% of the participants had depression, a proportion higher than the average estimated prevalence globally (Estimates, 2017). The risk factors for depression were also found, including women, lower income, and educational level. An existing study found risk factors for depression, which included low socioeconomic status (SES), women, and comorbid chronic medical conditions (i.e., obesity) (McCarron et al., 2021), consistent with the results of this study. A meta-analysis suggested that a low SES, ascertained by the use of proxies, such as education and income, was correlated with the risk of depression (Lorant et al., 2003). Previous studies have also confirmed that PA could generate benefits and reduce the risk of depression (Schuch et al., 2018; Dishman et al., 2021). Antidepressant mechanisms of PA proved that exercise could help reduce depression through biological mechanisms and psychosocial mechanisms (e.g., neuroplasticity, inflammation, oxidative stress, and the endocrine system in biological mechanisms and self-esteem, social support, as well as self-efficacy in psychosocial mechanisms; Kandola et al., 2019). However, in 2016, only 26% of men and 19% of women performed sufficient activities in the United States (Piercy et al., 2018). Thus, the participants without MVPA were found with a high proportion. Moreover, about 27.5% of participants did not meet the PA recommendation globally in 2016 (Guthold et al., 2018). Compared with the PA level globally, the low level of PA in this study led to a higher prevalence of depression than the average estimated prevalence worldwide.

Over the past few years, the correlation between PA level and the risk for depression has been extensively investigated, and persuasive evidence has been provided, showing that those with a higher level of PA had a lower risk for depression. More attention has been gradually paid to the intensity of PA. Several recent studies explored the correlation between PA intensity and the risk for depression. A large cross-sectional study conducted among 1.2 million US adults found that the mental health burden was reduced more significantly in those performing VPA (Chekroud et al., 2018), consistent with the results of this study. A higher proportion of VPA was inversely correlated with the lower risk for depression. There was no significance in a lower proportion, which was partly due to limited participants since a significant correlation was found in all proportion groups in multiple imputed data. Accordingly, an assumption was proposed that a higher proportion of VPA to MVPA may be confirmed in larger studies.

The protective effect of a high proportion of VPA to MVPA against depression was not confirmed in women and those aged less than 45 in this study. Currier et al. conducted the study among 13,884 Australian men and demonstrated that the respective additional hour of MPA replaced with VPA was correlated with a lower risk for depression (Currier et al., 2020). In comparison, Pavey et al. investigated the correlation between PA and the risk for depression among 11,285 Australian women and found no significantly additional benefits from VPA compared with MPA except at a very high PA level (Pavey et al., 2013). The above findings were consistent with the results of this study that men with a higher proportion of VPA had a lower risk for depression, and an insignificant correlation was observed between proportion and depression in women. Women were more likely to develop episodes due to certain unique subtypes of their depression (e.g., menarche, premenstrual dysphoric disorder, postpartum depression disorder, and perimenopausal depression; Angst et al., 2002; Kessler and Bromet, 2013). Moreover, men took more VPA (Barnekow-Bergkvist et al., 1996) and women primarily did much housework (Starmer et al., 2019). VPA may be more suitable for men but not for women. A previous study has even suggested that meeting either VPA or MPA recommendation was inversely correlated with a lower risk for depression in men but not in women (Asztalos et al., 2010). It may indicate that light-intensity PA was more suitable for women to prevent from depression. Another previous study has supported our assumptions and has found significant correlations between light-intensity PA and likelihood of depression only in women and between VPA and likelihood of depression only in men (Lindwall et al., 2007).

In the analysis stratified by age, this study found a significant correlation between proportion and depression in older adults but not in younger adults. A 2-year longitudinal cohort study found that older adults were more likely to suffer from depression (Schaakxs et al., 2018). In older adults, some factors might also increase the risk for depression, including single marital status (Markkula et al., 2016), social disconnectedness (Santini et al., 2020), and less support from family and society (Wang and Zhao, 2012). Compared with younger adults, PA could prevent more chronic diseases in older adults (e.g., cancer and cardiovascular disease). Moreover, older adults' mental health also could benefit more from PA relative to younger adults. The above benefits included social interaction and engagement (Zimmer et al., 2021). Joshi et al. ever investigated the effect of quantity and type of PA on subsequent depression among old adults, and found that those performing athletic activities were at a lower risk for depression (Joshi et al., 2016). It implied that maybe VPA could benefit more in older adults. Moreover, Lampinen et al. explored the correlation between physical intensity and depression among adults aged 65 and above with 8 years of follow-up, and found that depressive symptoms increased with a decreased intensity of PA (Lampinen et al., 2000). However, the perspectives regarding participation of older people varied: for some, physical activities were not necessary and even potentially harmful; however, others were aware of the benefits of PA but reported obstacles for PA participation (Franco et al., 2015). With aging, few activities were carried out among older adults (Vancampfort et al., 2017). To decrease the OR of depression, it was necessary to change the attitude toward PA among older people and improve access to PA participation.

This study investigated the correlation between proportion and the risk for depression and verified whether differences exist between subgroups separated by age and sex. It had several strengths. This study was conducted among large general participants, which would reveal the correlation more effectively. Moreover, the correlation between proportion and depression stratified by age and sex was estimated, which could find the differences between sex and age and provided the detailed suggestions for different sex and age. In addition, the correlation without considering the weight of intensity was analyzed besides analysis considering the weight of intensity. Furthermore, we also imputed the data and compared the analyses based on complete data and imputed data. Nonetheless, there were still some limitations in this study. Although our logistic regression model adjusted for many factors (e.g., age and sex), other confounding factors (e.g., genetic factors) were not adjusted. Furthermore, information regarding PA in this study was self-reported. Objective measurements of PA should be used to examine the volume of PA. Furthermore, this study was a cross-sectional study, which could not determine the direction of correlation or causal pathways. A randomized control trial could be implemented in further analysis.

## Conclusion

This study implied that a higher proportion of VPA to MVPA may be correlated with a lower risk for depression. However, the above findings may be only applied to men and older adults. No significant correlation was observed in women and younger adults. Men and older adults were suggested to perform a higher proportion of VPA, while suggestion may not be suitable for women and younger adults.

## Data Availability Statement

The datasets presented in this study can be found in online repositories. The names of the repository/repositories and accession number(s) can be found at: https://www.cdc.gov/nchs/nhanes/.

## Author Contributions

CY, DY, and MY: conception and design of the study. DY: collating data. DY and CY: analysis and/or interpretation of data. YM: visualization. DY, JB, and MY: writing the original manuscript. DY, CY, JB, YM, and MY: reviewing and editing the manuscript. CY: funding acquisition. All authors have read and agreed to the published version of the manuscript.

## Funding

This study was funded by the National Natural Science Foundation of China (Grant Nos. 82173626 and 81773552).

## Conflict of Interest

The authors declare that the research was conducted in the absence of any commercial or financial relationships that could be construed as a potential conflict of interest.

## Publisher's Note

All claims expressed in this article are solely those of the authors and do not necessarily represent those of their affiliated organizations, or those of the publisher, the editors and the reviewers. Any product that may be evaluated in this article, or claim that may be made by its manufacturer, is not guaranteed or endorsed by the publisher.
