# Supplementary material for: Association Between Physical Activity Intensity and the Risk for Depression Among Adults From the National Health and Nutrition Examination Survey 2007–2018
Source: Front Aging Neurosci. 2022 May 27;14:844414. doi: 10.3389/fnagi.2022.844414 (PMC9197339; doi:10.3389/fnagi.2022.844414)
Supplement: Supplementary file 1 [file Data_Sheet_1.pdf]

Supplementary materials

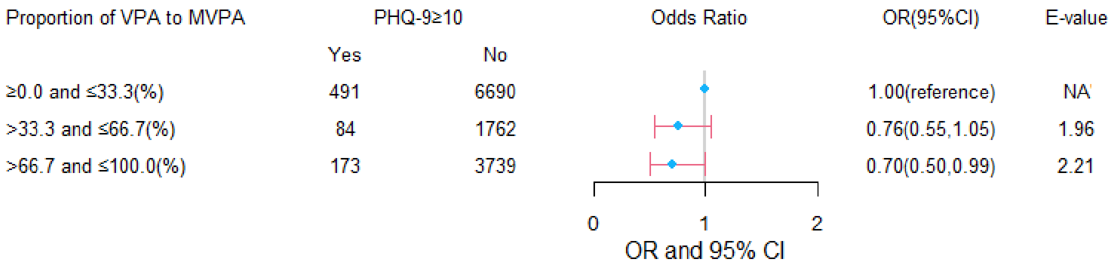

Abbreviations: VPA, vigorous physical activity; MVPA, moderate-to-vigorous physical activity; PHQ-9, Patient Health Questionnaire-9; OR, odds ratio; CI, confidence internal; NA, no applicable.

Fig. S1. E-value for correlation between the proportion of VPA to MVPA and the risk for depression. E-value means the minimum strength of correlation, on the risk ratio scale, that an unmeasured confounder would need to have with both the treatment and outcome, conditional on the measured covariates, to fully explain away a specific treatment–outcome correlation.

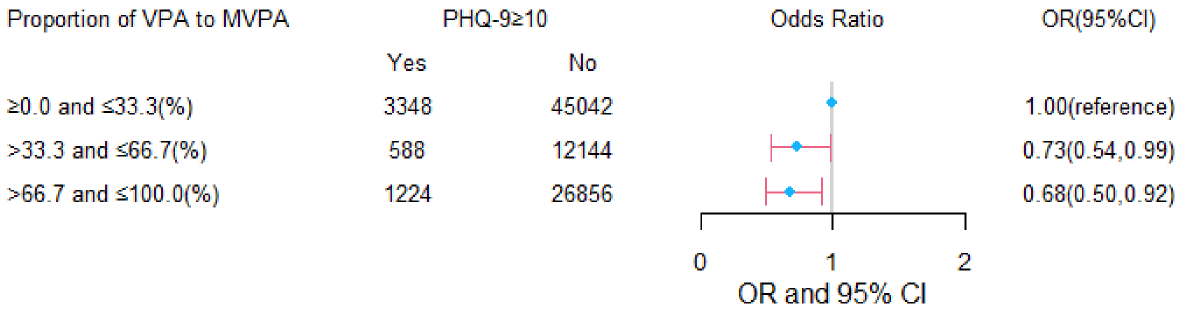

Abbreviations: VPA, vigorous physical activity; MVPA, moderate-to-vigorous physical activity; PHQ-9, Patient Health Questionnaire-9; OR, odds ratio; CI, confidence internal

Fig. S2. Correlation between proportion of VPA to MVPA and the risk for depression based on multiple imputed data.
